# Some Legal Points in Reference to Criminal Abortion

**Published:** 1898-07-16

**Authors:** 


### Medical Progress and Hospital Clinics.

[*The Editor will be glad to receive offers of co-operation and contributions from members of the profession. All letters should be addressed to* The Editor, at the Office, 28 & 29, Southampton Street, Strand, London, W.C.]

### SOME LEGAL POINTS IN REFERENCE TO CRIMINAL ABORTION.

By a Barrister-at-Law.

It is perhaps unnecessary to remind medical men of the existence of sections 58 and 59 of 24 and 25 Vict., c. 100, but we think there are some points in regard to criminal abortion which require explanation; and it is, therefore, our intention in this paper to state what those points are as briefly and concisely as is compatible with accuracy and completeness.

Practitioners are aware that the wording of section 58 of 24 and 25 Vict., c. 100, is as follows: "Every woman, being with child, who, with intent to procure her own miscarriage, shall unlawfully administer to herself any poison or other noxious thing, or shall unlawfully use any instrument, or other means whatsoever, with a like intent; and whosoever, with intent to procure the miscarriage of any woman, whether she be or be not with child, shall unlawfully administer to her, or cause to be taken by her any poison or other noxious thing, or shall unlawfully use any instrument, or other means whatsoever, with the like intent, shall be guilty of felony, and being convicted thereof shall be liable, at the discretion of the Court, to be kept in penal servitude for life, or for any term not less than five years (now three years), or to be imprisoned for any term not exceeding two years with or without hard labour, and with or without solitary confinement." Section 59 of the same statute enacts that, "Whosoever shall unlawfully supply or procure any poison, or other noxious thing or any instrument, or thing whatsoever, knowing that the same is intended to be unlawfully used or employed with intent to procure the miscarriage of any woman, whether she be or be not with child, shall be guilty of a misdemeanor, and being convicted thereof shall be kept in penal servitude for a term of three years, or be imprisoned for any term not exceeding two years, with or without hard labour."

So much, then, for the statutory enactments. We will now state the principles of law which have been decided from time to time in reference to the various points which have arisen on the wording of the two sections above mentioned.

In one case, A gave B a cake containing poison, which she merely put into her mouth and spat out again; in fact, she did not swallow any part of it. It was held that the mere delivery to the woman of this cake did not constitute an "administering" within the meaning of the statute, although the Judges seemed to think that swallowing it was not essential.[1] But, where the drug has been given for the purpose of procuring abortion, and it has been taken for that purpose in the prisoner's absence, this constitutes a "taking" within the statute.[2] The drug, however, must be a "poison" or a "noxious thing."[3] If the drug is a recognised poison, and given with an intention of producing abortion, although the dose is so small as to be incapable of doing any harm, this will constitute an "administering" with intent, &c.; further, where the drug is not poison, if the quantity administered is "noxious" the taking will amount to a felony.[4] It will not, however, when it is "innocuous," even when administered in large doses.[5] Proof must be forthcoming to show that there was intent to procure abortion. It is immaterial whether it was, in fact, likely or calculated to bring about a miscarriage.

Formerly (that is, prior to the year 1861) the woman must have been pregnant at the time, but since the passing of the Statute 24 and 25 Vict. c. 100, s.s. 58 and 59, it is not necessary that she should be so, the words used in the Act being "whether she be or be not with child." In another case a quill was used, and evidence was admitted to show that the prisoner had at other times caused miscarriages by similar means, and he was convicted.[6]

The words, "supplying a noxious thing," mean that the drug must be of a "noxious character"; it is not sufficient that being harmless in itself, it might, if taken, produce miscarriage by its mere action on the imagination.[7] At the same time, if the drug procures miscarriage, although there be no other evidence of its nature, this is sufficient evidence of its being a "noxious thing."[8] Again, if the drug supplied by the defendant be noxious, and he supplied it with intent to procure abortion, the offence is complete, although the woman herself may not have intended to use the drug, and although no other person than the defendant may have intended that it should be used for that purpose,[9] even if the woman was not, and never had been, pregnant.[10] It will be seen that under section 58 of the before-mentioned statute, if the woman with intent to procure her own miscarriage does any of the acts mentioned in that section, she only commits a crime under that section if she is with child, yet she may be convicted of conspiring with other persons to procure her own miscarriage, although she may not, in fact, be with child.[11] This, it may be noted, is the latest case on the subject of criminal abortion. Lastly, it is not necessary that the intention of employing the noxious drug should exist in the mind of any other person than the person supplying it.[12]

[1] R. v. Cadman, 1 Mood C.C., 114; [2] R. v. Wilson, 23 L.J. (M.C.), 18; [3] R. v. Isaacs, 32 L.J. (M.C.), 52; [4] R. v. Cramp, 5 Q.B.D., 307; [5] R. v. Hennah, 13 Cox, 547; [6] R. v. Dale, 16 Cox, 703; [7] See R. v. Isaacs, sup.; [8] R. v. Hollis, 12 Cox, 463; [9] R. v. Hillman, 33 L.J. (M.C.), 60; [10] R. v. Titley, 14 Cox, 502; [11] R. v. Whitchurch, 24 Q.B.D., 420 (1890); [12] See the case of the R. v. Hillman, sup.

